# Nonalcoholic fatty liver disease: molecular pathways and therapeutic strategies

**DOI:** 10.1186/1476-511X-12-171

**Published:** 2013-11-09

**Authors:** Yue Ye Huang, Aaron M Gusdon, Shen Qu

**Affiliations:** 1 Department of Endocrinology and Metabolism, Shanghai 10th People’s Hospital, School of Medicine, Tongji University, Shanghai 200072, China; 2 Department of Endocrinology and Metabolism, Nanjing Medical University, Nanjing 210029, Jiangsu Province, China; 3 Department of Neurology and Neuroscience, Weill Cornell Medical College, New York, NY 10065, USA

**Keywords:** Nonalcoholic fatty liver disease, Molecular mechanisms

## Abstract

Along with rising numbers of patients with metabolic syndrome, the prevalence of nonalcoholic fatty liver disease (NAFLD) has increased in proportion with the obesity epidemic. While there are no established treatments for NAFLD, current research is targeting new molecular mechanisms that underlie NAFLD and associated metabolic disorders. This review discusses some of these emerging molecular mechanisms and their therapeutic implications for the treatment of NAFLD. The basic research that has identified potential molecular targets for pharmacotherapy will be outlined.

## 

The prevalence of obesity is increasing worldwide. While there are no established treatments for NAFLD, however current research is targeting new molecular mechanisms that underlie NAFLD and associated metabolic disorders.

## Nuclear transcription factors: PXR and FXR

Studies in mice have shown that the Pregnane X Receptor (PXR), Farnesoid X Receptor (FXR), and Forkhead O1 are implicated in hepatic steatosis and in lipid and glucose metabolism [1]. It is accepted that nuclear transcription factors are major regulators of the metabolism of lipid and glucose but the specific pathways and interrelationships are still being investigated. PXR was initially found to activate transcription of steroid-inducible genes by forming a heterodimer with RXR and binding to PXR response elements in response to a number of different steroid precursors and metabolites as well as other both natural and synthetic compounds [2]. Many studies have demonstrated that PXR plays an important role in the clearance and elimination of toxic products of metabolism [3], however it has become clear that PXR also plays an important role in lipid metabolism as it was shown that PXR could increase fatty acid uptake by directly increasing the expression of the membrane bound fatty acid transporter (FAT, CD36) [4] PXR transgenic mice demonstrated hepatic steatosis [1]. Further studies have shown that PXR exerts its effect on lipid metabolism through several distinct mechanisms. In addition to its first known role in increasing fatty acid uptake into hepatocytes, PXR is also able to increase lipogenesis while also down regulating fatty acid beta oxidation. PXR’s ability to increase lipogenesis is due to enhanced transcription of stearoyl-CoA desaturase, fatty acid elongase, fatty acid synthase, and ATP citrate lyase [5]. These effects are largely due to the interaction of PXR with S14, which is response to various nutrient and hormonal signals [5]. The ability of PXR to downregulate fatty acid beta oxidation is largely due to its ability to impair signaling through the forkhead transcription factor Foxa2. Foxa2 induces transcription of genes necessary for fatty acid oxidation and ketogenesis and it is rendered inactive in the cytoplasm by insulin [6]. Foxa2 mediates the upregulation of carnitine palmitoyltransferase (Cpt1a, involved in beta-oxidation) and 3-hydroxy-3-methylglutarate-CoA synthase 2 (Hmgcs2, involved in ketogenesis), and PXR is able to directly bind Foxa2 and impair its ability to upregulate these genes [7]. Additionally, PXR has been shown to interact with the forkhead box-containing protein O subfamily (FOXO1). FOXO1 regulates the expression of several enzymes involved in gluconeogenesis and FOXO1 mRNA levels have been shown to correlate with the severity of NASH [8]. FOXO1 can act as a coactivator of PXR mediated transcription, however, their interaction is complex, and under the presence of the appropriate activator, PXR can act as a corepressor of FOXO1 mediated transcription [9].

Farnesoid X receptor (FXR) was originally described as a farnesol-activated receptor and has long been known for its function as a bile acid sensor in enterohepatic tissues [10]. FXR has emerged in recent years as a master regulator of lipid and glucose homeostasis in the liver and of inflammatory processes at hepatic and extrahepatic sites, and a number of synthetic FXR agonists are being tested for the treatment of different hepatic and metabolic disorders [11,12] given the ability of FXR to antagonize inflammatory and fibrogenic processes. Two FXR genes have been identified, FXRα and FXRβ. FXRα is expressed mainly in the liver, intestines, kidney, and adrenal glands, and at much lower levels in adipose tissues. FXRβ is a lanosterol sensor that encodes a functional protein in rodents but not in humans [13]. FXR is an obligate partner of the 9-cis-retinoic acid receptor RXR (retinoid X receptor). The relevance of FXR to hepatic physiology and disease became apparent from the observation that FXR-knockout mice on a high-fat diet exhibit hyperlipidemia and massive hepatic steatosis, as well as necroinflammation and fibrogenesis [14,15]. Importantly, a high fat diet led to macrosteatosis without inflammation in the livers of LDL receptor knockout mice whereas the livers of mice double-knockout (LDLR-/- and FXR-/-) mice showed necroinflammation and increased hepatic levels of tumor necrosis factor-α, intercellular adhesion molecule-1, transforming growth factor (TGF)–β, procollagen 1α1, and collagen, suggesting FXR may prevent progression of simple steatosis to NASH. FXR induces expression of genes that promote triglyceride clearance and mitochondrial fatty acid β-oxidation, as well as suppression of lipogenic gene transcription [16]. FXR also upregulates the genes encoding apolipoprotein C-II (apoC-II) and very-low-density lipoprotein receptor, therefore enhancing triglyceride-rich lipoprotein clearance, while apoA-I gene expression is suppressed [17]. Synthetic FXR agonists decrease gluconeogenesis in mice fed a high-fat diet by reducing the expression and activity of PEPCK, G6Pase, and fructose-1,6-biphosphatase, and by enhancing insulin signaling through insulin receptor substrate-1 (IRS-1) phosphorylation [18,19]. FXR also appears to antagonize hepatic inflammatory and fibrogenetic processes. Another hepatic protective mechanism of FXR activation has been shown to be maintenance of gut integrity against gut-derived endotoxins through induction of antibacterial factors such as angiogenin, iNOS, and interleukin (IL)–18 [20].

### PPARs, RXR and FGF21

Three distinct members of the peroxisome proliferator activated receptor (PPAR) sub-family each encoded by a distinct gene have been identified and well characterized. PPARα (NR1C1) is highly expressed in liver, kidney, and muscle. PPARγ (NR1C3) is enriched in adipose tissue and PPARβ/δ (NR1C2; referred to as PPARδ in this review) appears to be ubiquitously expressed. All three PPARs bind to DNA as heterodimers with retinoid X receptors (RXR; NR2B sub-group) and bind preferentially to direct repeats of the nuclear receptor half site AGGTCA septed by 1 nucleotide (DR1). Each sub-type appears to have unique functions and PPARα and PPARγ are the targets of the fibrate and thiazolidinedione (TZD) classes of drugs respectively.

Studies in vitro and in vivo have demonstrated that PPARα directly regulates a network of genes encoding the proteins required for the uptake of fatty acids, enzymes required of the oxidation of fatty acids, and enzymes required for ketogenesis by binding to control regions in the promoter of these genes. Thus, activation of PPARα promotes the utilization of fat as an energy source. Mice lacking PPARα accumulate triglycerides in the liver and become hypoglycemic during fasting or starvation. Recent studies indicate that fibroblast growth factor 21 (FGF21) functions as an endocrine hormone that mediates many of the effects of PPARα. The gene encoding FGF21is directly induced by PPARα in response to fasting via a binding site in the promoter. FGF21 in turn stimulates lipolysis in adipose tissue and ketogenesis in the liver. In vitro studies have demonstrated that fatty acids can bind to PPARα and function as ligands. More recently, affinity purification of PPARα from mouse livers followed by mass spectrometery identified 1-palmityol-2-oleoyl-sn-glycerol-3-phosphocholine (16:0/18:1-GPC) as a selective PPARα agonist [21]. Interestingly, the production of 16:0/18:1-GPC requires the activity of fatty acid synthase (FAS) suggesting that PPARα-dependent fat oxidation serves as a regulatory mechanism to ensure that fatty acids levels stay within an optimum range. Taken together PPARα appears to function as a sensor of the fed/starved state.

PPARγ is a master transcriptional regulator of adipogenesis and plays an important role in the process of lipid storage [22]. Thus PPARα and PPARγ have opposing functions in the regulation of fat metabolism; PPARα promotes utilization while activation of PPARγ promotes storage. Indeed, it has been suggested that PPARγ’s role in the activation of lipogenic genes may contribute to the development of steatosis, as increased PPARγ expression has been found in steatotic livers. Nevertheless, several studies have shown that overexpression of PPARγ can prevent the progression of hepatic steatosis in mouse models and treatment with the PPARγ agonist rosiglitazone has been shown to have similar effects. The protective effects of PPARγ could be due to increased insulin sensitivity in adipose tissue and skeletal muscle leading to decreased FFA deposition in the liver. Adiponectin has also been shown to be increased by PPARγ which also contributes to insulin sensitivity as well as up-regulating PPARα expression, which leads to further hepatic fatty acid oxidation. Furthermore, PPARγ expression has been shown to have anti-inflammatory and anti-fibrotic effects in stellate cells, macrophages, and epithelial cells.

A number of naturally occurring fatty acids and prostanoids have been shown to act as PPARγ agonists, however, perhaps most importantly was the identification that the TZD class of insulin sensitizing drugs including rosiglitazone (Avandia) and pioglitazone (Actos) are PPARγ agonists. Fatty acid accumulation in insulin sensitive tissues such as liver and skeletal muscle has been shown to promote insulin resistance. Activation of PPARγ in adipose has been proposed to increase the number of adipocytes and promote the relocalization and storage of fat in adipose tissue, protecting peripheral tissues from lipotoxicity. Consistent with this idea is the observation that selective knockout of PPARγ in adipose tissue eliminates the therapeutic activity of TZDs in mice and that a common side effect of TZD in treatment in humans is weight gain due to an increase in adipose mass. The TZDs have proven to be effective drugs for improving insulin sensitivity and treating type II diabetes. Nevertheless, they are not without problems. Troglitazone, the first TZD to be used in the clinic, was taken off the market because of cases of drug-induced liver damage. Additionally recent meta-analyses have indicated that treatment with rosiglitazone is associated with increased risk of myocardial infarction and deaths due to cardiovascular events [23]. Pioglitazone treatment was also shown to be associated with an increase in heart failure although a significantly lower risk of myocardial infarction and death was observed in this patient population. Finally, muraglitazar an investigational drug that is a dual agonist of PPARα and PPARγ was found to be associated with an increase in major cardiovascular events and increased incidence of death. The increases in cardiovascular events and mortality seen with these drugs are relatively small. Nevertheless, the wide scale use of PPARγ agonists in the type II diabetic population has raised serious concerns about the safety of these drugs for long term therapy. The molecular basis underlying the increase in cardiovascular events is not clear. Regulation of the epithelial sodium channel (ENaCγ) in the kidney by PPARγ has been suggested as potential mechanism responsible for TZD-dependent edema but it remains to be seen if inhibiting this channel will decrease the cardiovascular events associated with TZD treatment. As the peroxisome proliferator-activated receptors (PPARs) represent major regulators of lipid metabolism in the liver, a few studies have tested the hypothesis that genetic variants in these hormone receptors may influence susceptibility to NAFLD, but the results have been controversial [24-26]. As PPARs are also the target of several drugs under evaluation for the treatment of NAFLD, this evidence may lay the basis to design pharmacogenetic studies to assess the role of PPAR SNPs in predicting the response to drugs targeting these nuclear receptors.

### Pex11a

Peroxisomes are organelles which have important roles in cellular metabolism including very-long chain fatty acid β-oxidation, cholesterol synthesis, and hydrogen peroxide metabolism [27-29]. Peroxisome proliferation is dependent on Pex11, which has been shown to promote peroxoisome elongation in various model systems [30-34]. Three subtypes of Pex11 have been identified (α, β, and γ), with Pex11α and γ being primary expressed in the liver and Pex11β being more broadly expressed [34,35]. Weng et al. [36] recently demonstrated that mice with Pex11α knocked out had increased body weight and accumulated fat in their livers. Pex11α knockout mice exhibited elevated levels of free fatty acids without significant effects on glucose or beta-hydroxybutyrate. While loss of Pex11α impaired peroxisomal elongation, it also decreased the expression of genes important for peroxisomal fatty acid oxidation. Therefore, targeting peroxisomal function may represent a new therapeutic target in the treatment of NAFLD.

### P53

P53 is well known to be a key regulatory of cellular metabolism and has been shown to regulate the balance between oxidative and glycolytic metabolism in cancer cells [37]. Recently, p53 has also been implicated in NAFLD largely through its role in regulating fatty acid oxidation. Similar to its role in alcoholic fatty liver disease, p53 has been shown to be upregulated in the livers of mice with NAFLD [38] and has been linked to hepatocytes apoptosis in NAFLD [39]. To test the viability of p53 as a therapeutic target in NAFLD, Derdak et al. looked at the effect of a p53 inhibitor, pifithrin-αp-nitro (PFT) on various pmeters of NAFLD in a murine model [40]. In HFD fed C57BL/6 mice, PFT treatment was found to reduce hepatic steatosis, apoptosis, and oxidative stress. These effects are believed to be secondary to a reduction in the upregulation of miRNA34a and a shift toward high activity of the SIRT1/AMPK/ACC pathway. SIRT1 increases the expression of AMPK which blocks ACC allowing CPT1 to be activity due to reduced levels of the inhibitory malonyl-CoA. In another arm of the pathway, SIRT1 is also believed to upregulate PGC1α/PPARα, which upregulates MLYCD subsequently decreasing MLYCD levels, decreasing Malonyl-CoA synthesis and relieving the repression of CPT1. Another recent study confirmed miR-34a upregulation and involvement of SIRT1 and p53 in liver steatosis and found that treatment with ursodeoxycholic acid reversed p53 upregulation and restored SIRT1 expression [41]. Taken together, these results support the role of p53 in regulating metabolism and suggest that it may be a target for treatment of NAFLD.

### Liver X receptor

The Liver X receptor (LXR) sub-group of the nuclear receptor superfamily is comprised of two sub-types, LXRα and LXRβ that are encoded by septe genes. LXRα was originally cloned from a liver cDNA library and found to be highly expressed in the liver, kidney, and intestine. In contrast, LXRβ is more ubiquitously expressed. LXRs are important regulators of cholesterol and lipid metabolism, and their activation has been shown to inhibit cardiovascular disease and reduce atherosclerosis in animal models. More recent studies using knockout mice deficient in the synthsis of oxysterols have provided strong support that these cholesterol derivatives function as LXR ligands in vivo [42]. The identification of hydroxycholesterols as natural LXR ligands prompted studies to identify the genetic networks regulating cholesterol metabolism which are controlled by LXR. An increase in hepatic cholesterol levels is predicted to lead to an elevation in the concentration of cholesterol-derived LXR ligands resulting in the catabolism of cholesterol to bile acid and the excretion of cholesterol out of the liver. Indeed, when challenged with a diet rich in cholesterol Lxrα-/- mice accumulate massive amounts of cholesterol in the liver. In mice, pharmacological activation of LXR has been shown to result in net movement of cholesterol out of the body, a result consistent with the gene expression and genetic knockout studies. In concert with the elimination of hepatic cholesterol, activation of LXR also decreases cholesterol uptake by inducing expression of the Inducible Degrader of the LDL receptor (Idol) [43]. Over expression of Idol in cell culture models significantly enhances the ubiquitination and proteosome-dependent degradation of the LDL receptor. Additionally, Idol is expressed in macrophages where it may also affect cholesterol uptake.

Along with effects on cholesterol metabolism activation of LXR agonists also increases expression of genes involved in fatty acid metabolism including the master transcriptional regulator of fatty acid synthesis, sterol response element binding protein 1c (SREBP1c) [1]. Several of the genes encoding the enzymes involved in fatty acid metabolism including fatty acid synthase (FAS) and stearoyl CoA desaturase 1 (SCD-1) are directly or indirectly regulated by LXR. The up-regulation of fatty acid synthesis in coordination with reverse cholesterol transport is most likely to provide lipids for the transport and storage of cholesterol. Small molecule agonists of LXR activity are therefore of great therapeutic interest. Nevertheless, the elevations in plasma cholesterol and triglyceride levels seen in LXR agonist-treated animals have severely limited the potential transition of the first generation LXR agonists into the clinic. However, the finding that such agonists also promote hepatic lipogenesis has led to the idea that hepatic LXR activity may be undesirable from a therapeutic perspective. Liver-specific deletion of LXR eliminated the detrimental effect of increased plasma triglycerides, while the beneficial effect of increased plasma HDL was unaltered.

There are numerous studies that have reported LXR plays a major role in the regulation of macrophage reverse cholesterol transportation (RCT) and that will contribute to the lipid accumulation in the liver and development of atherosclerosis. The Liver X Receptors (LXRs) α and β and the Peroxisome Proliferator-Activated Receptor α (PPARα) are transcription factors that belong to class II nuclear receptors. They drive the expression of genes involved in hepatic lipid homeostasis and therefore are important targets for the prevention and treatment of nonalcoholic fatty liver disease (NAFLD). LXRs and PPARα are regulated by endogenous ligands, oxysterols and fatty acid derived molecules. In the liver, pharmacological activation of LXRs leads to the over-expression of genes involved in de novo lipogenesis, while activation of PPARα is critical for fatty acid catabolism in nutrient deprivation. While these two nuclear receptors appear to have opposing effects, recent studies have highlighted that PPARα also influences the expression of genes involved in fatty acids synthesis. T0901317 and fenofibrate induce expression of genes involved inLXR-dependent and PPARα-dependent lipogenic responses. Deficiency of PPARα does not influence the effects of T0901317 on lipogenic genes expression. However, PPARα deficiency prevents the up-regulation of genes involved in ω-hydroxylation that are induced by T0901317. In addition, over-expression of lipogenic genes in response to fenofibrate is decreased in LXR knockout mice as well as the expression of PPARα target genes involved in fatty acid oxidation. This supports the existence of an interaction between these nuclear receptors and suggests LXR and PPARα may have overlapping control over the expression of hepatic genes involved in lipid metabolism.

### Thyroid hormone receptor

The antiobesity and low-density lipoprotein (LDL)–cholesterol–lowering effects of thyroid hormones have been known for years, but their pharmacological use has been limited by their untoward effects on bone and heart and skeletal muscle. Recent research has developed compounds that lack these adverse effects while harnessing the beneficial effects of thyroid hormone. The development of these drugs, termed thyromimetics, was made possible by the existence of distinct thyroid hormone receptor (TR) subtypes with different tissue-specific distributions [44]. The two subtypes of TRs, TRα and TRβ, are predominantly localized in the heart and liver, respectively. Selective TRβ1 agonists have been synthesized and evaluated in preclinical trials and in a small clinical trial, demonstrating their lipid-lowering properties and safety [45]. The liver specificity of these drugs is achieved through the selective hepatic distribution of phosphonic acid: which is phosphonate-containing TRβ1 agonist that is taken up predominantly by the liver with a high first pass extraction and converted into the active form in hepatic microsomes by cytochrome P450 3A. Recently, the main compound of this type, MB07811, has been evaluated in different animal models of hepatic steatosis, including ob/ob mice, Zucker diabetic rats, and mice with diet-induced obesity [46]. A marked reduction was observed in plasma cholesterol and triglyceride levels. Hepatic selectivity of MB07811 was demonstrated by showing that this agent did not affect circulating FFA levels, epidydimal fat pad and heart mass, or pituitary thyroid-stimulating hormone mRNA. A decrease in hepatic lipogenesis, as suggested by a reduction in SREBP-1c mRNA, and in hepatic lipase activity, as suggested by reduced levels of apolipoprotein C3 (apo C3, an inhibitor of lipoprotein lipase), might also contribute to the antisteatotic effects of this agent. GC-1, another TRβ1-selective activator, ameliorated not only steatosis but also markers of hepatic lipoperoxidation and liver injury, suggesting these agents have the potential to reverse not only fat infiltration but also the progressive features of steatohepatitis. How TR mimetics can ameliorate hepatocyte oxidative stress and necroinflammation warrants further investigation. These futile cycles reduce oxidative stress in two ways: they increase ATP cycling, which enhances oxidative phosphorylation and decreases the formation of reactive oxygen species (ROS); and they supply the NADPH necessary for the regeneration of reduced glutathione (GSH) and other reducing substances, thus facilitating the scavenging of ROS [47]. Collectively, the results of the studies show promise for the treatment of NAFLD and associated metabolic disorders, but efficacy and safety of this class of agents in humans need further evaluation.

### AMP-activated protein kinanse (AMPK)

As discussed in previous sections, signaling through AMP can have a beneficial effect on hepatic steatosis. AMPK has emerged as a central regulator of cellular metabolism. AMPK consists of heteromeric complexes with a catalytic α subunit and regulatory β and γ subunits. AMPK is able to sense the energy status of the cell in a variety of different ways. The γ subunits are able to bind adenine nucleotides, with ADP and AMP leading to activation of AMPK though allosteric activation and phosphorylation [48]. AMPK is able to regulate metabolism through its effects on glucose homeostasis, lipid metabolism, protein synthesis, and oxidative metabolism. AMPK exerts its control over glucose metabolism at a number of different steps in glucose utilization. AMPK promotes the upregulation of the proteins needed for glucose uptake into cells [49], phosphorylates 6-phosphofructo-2-kinase (PFK-2) thereby upregulating glycolysis [50], inhibiting gluconeogenesis though multiple mechanisms [51], and phosphorylating and inhibiting glycogen synthase and subsequently reducing the amount of glycogen synthesized [52]. AMPK is able to control lipid metabolism by both decreasing lipogenesis and stimulating mitochondrial fatty acid oxidation. The former task is accomplished by phosphorylating 3-hydroxy-3-methylglutaryl-coenzyme A reducase (HMG-CoA) and acetyl CoA carboxylase 1 (ACC1). Inactivation of HMG-CoA reducase blocks the conversion of HMG-CoA to mevalonate and inactivation of ACC1 results in decreased ability to synthesize fatty acids. AMPK’s ability to promote fatty acid oxidation is attributed to decreased synthesis of malonyl CoA due to phosphorylation of ACC2 which relieves the inhibition of carnitine O-palmitoyltransferase 1 (CPT1). Recently, AMPK has also been shown to phosphorylate the transcriptional co-activator transducer of regulated CREB activity 2 (TORC2), excluding it from the nucleus and decreasing transcription of the gluconeogenic genes PEPCK and glucose-6-phosphatase [53]. Importantly, AMPK has also been shown to decrease transcription of lipogenic genes thought its inhibition of sterol regulatory element-binding protein-1c (SREBP1c) [54]. Additionally, AMPK has been shown to upregulate oxidative phosphorylation through its ability to positively regulate PGC-1α through phosphorylation, which subsequently increases mitochondrial biogenesis [55].

Given its ability to sense and regulate the metabolic state of the cell, modulation of AMPK has become an important therapeutic target in NAFLD. Exercise has well known beneficial effects in the metabolic syndrome as well as in NAFLD and some of these effects may be due to its modulation of the AMPK signaling pathway as expression of genes induced by exercise requires AMPK activation [56,57]. Various compounds already in use for the treatment of type 2 diabetes have been shown to have effects on the AMPK pathway. The beneficial effects of thiazolidinediones on hepatic steatosis were discussed above with activation of PPARα as a driving mechanism. However, the thiazolidinediones have also been shown to increase the AMPK signaling pathway with improvement in both hepatic steatosis and fibrosis [58]. Additionally, biguanides such as metformin have been shown to induce AMPK activity in hepatocytes subsequently increasing fatty acid oxidation, decreasing lipogenesis, and decreasing hepatic glucose production [59]. Treatment with metformin has been shown to reverse liver steatosis and its lipid lower effects were shown to be dependent on AMPK activation [60]. The mechanism by which AMPK is induced by both of these classes of drugs likely involves alteration of mitochondrial function. Thiazolidinediones bind to and impair assembly of complex I [61] as well as impairing mitochdrial pyruvate transport [62] and metformin has been shown to inhibit mitochondrial complex I thus decreasing the ATP to ADP/AMP ratio and inducing AMPK.

Polyphenols such as resveratrol [63] and epigallocatechin gallate [64] have been demonstrated to activate AMPK. One suggested mechanism has been the ability of these compounds to activate sirtuin 1 (SIRT1), which acts as a metabolic regulatory through its ability to sense NAD + levels and also is an upstream regulator of AMPK [65]. Epigallocatechin gallate’s may also increase reactive oxygen species production, and subsequently activate CaMKK which can phosphorylate AMPK [66]. However, these compounds also likely act through their effects on adenine nucleotide levels as resveratrol has been shown to inhibit mitochondrial complex V [67] and epigallacatechin gallate has been shown to inhibit mitochondrial complexes I, II, and V [68].

5-aminoimidazole-4-carboxamide-1-b-d-ribofuranoside (AICAR) has been extensively demonstrated to activate hepatic AMPK activity. Treatment with AICAR has been shown to decease hepatic lipid accumulation through AMPK activation [69] and also decrease hepatic glucose production largely through TORC2 inactivation as well as increasing skeletal muscle glucose uptake [70]. While AICAR’s effects on AMPK is due to allosteric activation by its metabolite 5-aminoimidazole-4-carboxamide ribotide (ZMP) which is structurally similar to 5’-AMP, it was originally believed that AICAR treatment did not affect endogenous adenine nucleotide levels. However, recent studies have shown that AICAR treatment results in ATP depletion [71] and can impair mitochondrial function [72], potentially limiting its therapeutic use.

More recently, other activators of AMPK have been discovered after screening chemical libraries. A-769662 has been found to increase AMPK activity through allosteric activation as well as inhibition of dephosphorylation [73]. Furthermore, A-769662 treatment did not alter adenine nucleotide levels nor affect mitochondrial function [74].

Betaine is a metabolite of choline and has previously been shown to be beneficial in alcoholic liver disease. Given its ability to activate AMPK [75], it has also been sought as a treatment for NAFLD. Indeed, an initial pilot study reported beneficial effects of betaine treatment on various pmeters of liver function including reduced steatosis and fibrosis.

α-Lipoic acid has been shown increase glucose uptake by skeletal muscle and also to activate AMPK in resulting in increased fatty acid oxidation in skeletal muscle [76]. Additionally, part of α-Lipoic acid’s beneficial effects may be due to activation of PPARα and FGF21 [77].

AMPK is also modulated by hormones and cytokines such as resistin, leptin, adiponectin, and ghrelin [53]. The ability of adiponectin to lower hepatic glucose production has been attributed to activation of AMPK [78], as adiponectin was unable to regulate hepatic glucose production in AMPKα2 mice [79]. Adiponectin has been shown to decrease hepatic triglyceride content and thereby restore insulin sensitivity and decrease hepatic fat deposition [80].

### Bile-acid receptor and TGR5

Bile acids (BAs) are critical regulators of hepatic lipid and glucose metabolism and signal through two major receptor pathways: FXR (as discussed above) and TGR5, the G protein-coupled bile acid receptor (GPBAR1). Both FXR and TGR5 demonstrate pleiotropic functions including immune modulation. The activation of BA receptors modulates hepatic monocyte activity and improves non-alcoholic fatty liver disease. McMahanet al [81] reported that obese db/db mice were treated with a dual FXR/TGR5 agonist (INT-767) for 6 weeks and the histologic features of NASH in these mice were significantly improved. Furthermore, treatment increased the proportion of intrahepatic monocytes with the anti-inflammatory Ly6Clow phenotype and increased intrahepatic expression of genes expressed by alternatively activated macrophages. In vitro treatment of monocytes with INT-767 led to decreased Ly6C expression and increased IL-10 production through a cAMP-dependent pathway. This indicates that FXR/TGR5 activation may be an important therapeutic target in NAFLD by coordinating the immune phenotype of both monocytes and macrophages.

### Nrf2

Nuclear erythroid 2-related factor 2 (Nrf2) is an oxidative stress-mediated transcription factor with a variety of downstream targets aimed at cytoprotection. Nrf2 has recently been implicated as a new therapeutic target for the treatment of liver disease. Lipid peroxidation has been shown to impair nucleotide and protein synthesis, thereby inducing apoptosis, inflammation, and liver fibrosis. It has been proposed that Nrf2 plays a role in NASH because genetic deletion of Nrf2 in mice results in rapid onset and progression of the disease. There are several stages of disease pathogenesis on which activation of Nrf2 may exert a potential therapeutic effect. The first is in the initial stages of the disease, when lipids are accumulating within the hepatocytes. Nrf2 activation with CDDO-Im has been shown to effectively prevent hepatic lipid accumulation in wild-type mice but not in Nrf2- deficient mice, an effect that may be mediated by a decrease in expression of enzymes required for fatty acid synthesis. Nrf2 may also protect against NAFLD by decreasing inflammation. Several chemotherapeutic agents have been shown in a variety of cell culture and rodent systems to induce Nrf2 and cause simultaneous repression of nuclear factor-κB (NF-κB), an important cell-signaling molecule for the inflammatory response. Additionally, pharmacological activation of Nrf2 results in increased gene expression of Heme oxygenase 1(Ho-1) and NAD(P)H quinone oxidoreductase 1 (Nqo1), which in turn have inhibitory effects on inflammation [82]. Ho-1 has been shown in mice to prevent phosphorylation of NF-κB by its endogenous substrate tumor necrosis factor-α, 61 indicating that it has inhibitory effects upstream on the initiation of the inflammatory response. Under normal conditions, lipopolysaccharide(LPS) can be used to induce the expression of tumor necrosis factor-α and cause inflammation through activation of NF- κB, however, Nqo1 and Ho-1 overexpression, such as occurs with Nrf2 activation, has been shown in human monocytes to prevent the LPS mediated induction of tumor necrosis factor-α expression, thereby preventing inflammation. Another mechanism for the potential treatment of NAFLD and NASH is through activation of superoxide dismutase and catalase, antioxidant enzymes with decreased activity in this disease state. The pharmaceutical agent and Nrf2 activator Protandim has been shown in human trials to increase erythrocyte superoxide dismutase and catalase activity by 30 and 54%, respectively. Finally, it is possible that activation of Nrf2 could play an inhibitory role on transforming growth factor-β (TGF-β), a profibrotic signaling factor in plasma, and therefore may inhibit the progression of fibrosis in NASH. A recent study demonstrated that sulforaphane attenuates hepatic fibrosis through Nrf2-mediated inhibition of TGFβ signaling in a human hepatic stellate cell line. This effect was ultimately due to the suppression of hepatic stellate cell activation and fibrogenic gene expression, indicating that activation of Nrf2 has an antifibrotic effect in liver. These findings support a role for Nrf2 signaling in various steps of the pathogenesis of NAFLD.

### Mitochondria

Given their central role in metabolism and fatty acid homeostasis, much attention has recently been focused on the role of mitochondrial function in NAFLD and NASH. However, the exact role of mitochondrial function remains far from clear, and several studies have yielded conflicting results. While a detailed over of all of the studies on the topic is beyond the scope of this review and has been reviewed in detail elsewhere [83-85], we will present a brief overview here. In healthy individuals, lipid uptake and synthesis is relatively well balanced with fatty acid oxidation and lipid export as VLDL being matched by lipid synthesis and lipid uptake [86]. However, insulin resistance and hyperglycemia lead both to increased lipolysis in white adipose tissue which increases the serum fatty acid concentration as well as increased lipid synthesis and uptake by the liver resulting in fat accumulation [87]. Liver mitochondria exhibit the ability to adapt to these changing metabolic conditions and the majority of studies indicate that in various states of disease progression, mitochondria are able to increase fatty acid oxidation in an attempt to counteract liver fat accumulation [88-90]. Various mechanisms underlie this increase in fatty acid oxidation including higher levels of leptin [91], FGF21 [92,93], and IL-6 [94]. PPARα activation has also been demonstrated [95,96] and can upregulate several genes involved in fatty acid oxidation namely CPT1 [97]. While fatty acid oxidation is able to be upregulated, most of the available evidence has shown that ATP levels are lower in patients with NASH as well as in animal models [98] even though a number of studies have demonstrated that overall oxygen consumption is either increased [99] or unchanged [100]. These seemingly pdoxical results may be due to increased expression of UCP2 in NAFLD resulting in mitochondrial uncoupling [101]. This may well represent a potentially adaptive mechanism to limit reactive oxygen species production which would be presumably induced by the influx of reducing equivalents from increased fatty acid oxidation [102]. However, this mechanism is seemingly insufficient to protect mitochondria from free radical mediated damage as impaired enzymatic activity of various complexes of the electron transport chain has been repeatedly demonstrated [103,104]. Several electron transport chain complexes have been shown to be susceptible to inhibition by ROS. ROS can also led to lipid peroxidation which can induce dysfunction of complex IV and the adenine nucleotide translocator as well as altering mitochondrial DNA [105]. Indeed, as increased fatty acid oxidation results in more ROS production and damage to the electron transport chain, a further mismatch between the two likely occurs presumably resulting in progressively more electrons being lost as free radicals [102].

Therapeutic strategies to target mitochondria as treatments for NAFLD and NASH are still in the very early stages. Approaches to further increase fatty acid oxidation have been proposed in order to decrease the accumulation of fat in the liver [106], however this approach may further exacerbate the imbalance between fatty acid oxidation and the electron transport chain further increasing ROS production. To this point, inhibition of CPT-I, which catalyzes the rate-limiting step of fatty acid oxidation, has been shown to prevent high fat diet induced insulin resistance, in part due to a reduction in some of the deleterious intermediates generated by incomplete fatty acid oxidation and also due to a shift toward increased glucose oxidation for energy production [107]. Therefore, the development of viable therapeutics targeting mitochondrial pathways will likely be more complicated than anticipated and should take into account not only the effect on the liver but also potential effect on the metabolic pathways of other tissues. Nevertheless, some compounds shown to improve mitochondrial function have shown initial promise in treating NAFLD.

Curcumin, which is known to have hepatoprotective effects, was shown to inhibit the progression of hepatic steatosis in a rabbit model by improving mitochondrial respiratory chain function, decreasing oxidative stress, and reducing levels of TNF-α [108]. Similarly, silybin, which has antioxidant, anti-inflammatory, and anti-fibrotic activity, has been shown to protect against oxidative stress, preserve mitochondrial function, and limit the progression of NASH in an animal model [109]. Given their already widespread use and minimal side effect profile, other antioxidants such as vitamin E, vitamin C, and N-acetylcysteine have been studied in models of NASH with favorable outcomes [110]. While extensive clinical data demonstrating the beneficial effect of antioxidants in NASH remain to be generated, early studies have shown promise. For instance, a silybin-vitamin E compound has been shown to improve various pmeters of liver function in patients with NAFLD.

### Cannabinoid receptors

Cannabinoids exert their effects through two different cannabinoid receptors: CB1 and CB2, both of which are G-protein coupled. Both of these receptors have been implicated in the development of liver fibrosis secondary to various etiologies. It has been shown that marijuana use may correlate with the progression of liver fibrosis in patients with hepatitis C [111]. However, each receptor seems to have opposing roles in the liver. The rCB2 recepto has been shown to be up-regulated in the livers of cirrhotic patients and has been shown to ameliorate the progression of fibrosis [112]. In contrast, CB1 receptor activation has been linked to the progression of fibrosis and CB1 antagonists have been shown to inhibit the progression of fibrosis [113]. Indeed, clinical trials with a CB1 receptor antagonist have shown that antagonism of CB1 can result in weight loss and improved metabolic and cardiac pmeters in overweight and obese populations [114]. However, given considerable side effects, future compounds developed for the treatment of NASH will likely need to be peripherally targeted [115].

Cannabinoid receptors are have emerged as major targets for the treatment of obesity. New compounds which target cannabinoid receptors have been developed for weight loss and may prove to be efficacious in the management of NAFLD. Experimental and clinical data indicate that peripheral activation of cannabinoid CB1 receptors promotes insulin resistance and liver steatogenesis, two key steps in the pathogenesis of non-alcoholic fatty liver disease. Moreover, activation of CB1 receptorsenhance progression of liver fibrogenesis. These findings provide the rationale for the use of CB1 antagonists in the management of NAFLD or NASH.

## Conclusions

In this review, we have outlined some of the molecular pathways contributing to the pathogenesis of NAFLD. While the details of many of these pathways are still being elucidated, they provide targets for future therapeutic strategies in NAFLD. Still, much work remains to be done in detailing the exact mechanisms contributing to NALD and the coordination and interaction among the various pathways that have already been implicated in disease pathogenesis.

## Competing interests

The authors declare that they have no competing interests.

## Authors’ contributions

SQ wrote the manuscript; YYH provided all the reference and also wrote the manuscript; AMG was involved in editing the manuscript. All authors read and approved the final manuscript.

## References

[B1] Zhou J Hepatic fatty acid transporter Cd36 is a common target of LXR, PXR, and PPARgamma in promoting steatosis Gastroenterology 2008 134 2 556 567 10.1053/j.gastro.2007.11.037 18242221

[B2] Blumberg B SXR, a novel steroid and xenobiotic-sensing nuclear receptor Genes Dev 1998 12 20 3195 3205 10.1101/gad.12.20.3195 9784494 PMC317212

[B3] Timsit YE Negishi M CAR and PXR: the xenobiotic-sensing receptors Steroids 2007 72 3 231 246 10.1016/j.steroids.2006.12.006 17284330 PMC1950246

[B4] Zhou J A novel pregnane X receptor-mediated and sterol regulatory element-binding protein-independent lipogenic pathway J Biol Chem 2006 281 21 15013 15020 10.1074/jbc.M511116200 16556603 PMC4109972

[B5] Moreau A A novel pregnane X receptor and S14-mediated lipogenic pathway in human hepatocyte Hepatology 2009 49 6 2068 2079 10.1002/hep.22907 19437491

[B6] Wolfrum C Asilmaz E Luca E Friedman JM Stoffel M Foxa2 regulates lipid metabolism and ketogenesis in the liver during fasting and in diabetes Nature 2004 432 7020 1027 1032 10.1038/nature03047 15616563

[B7] Nakamura K Moore R Negishi M Sueyoshi T Nuclear pregnane X receptor cross-talk with FoxA2 to mediate drug-induced regulation of lipid metabolism in fasting mouse liver J Biol Chem 2007 282 13 9768 9776 10.1074/jbc.M610072200 17267396 PMC2258557

[B8] Valenti L Increased expression and activity of the transcription factor FOXO1 in nonalcoholic steatohepatitis Diabetes 2008 57 5 1355 1362 10.2337/db07-0714 18316359

[B9] Kodama S Koike C Negishi M Yamamoto Y Nuclear receptors CAR and PXR cross talk with FOXO1 to regulate genes that encode drug-metabolizing and gluconeogenic enzymes Mol Cell Biol 2004 24 18 7931 7940 10.1128/MCB.24.18.7931-7940.2004 15340055 PMC515037

[B10] Parks DJ Bile acids: natural ligands for an orphan nuclear receptor Science 1999 284 5418 1365 1368 10.1126/science.284.5418.1365 10334993

[B11] Downes M A chemical, genetic, and structural analysis of the nuclear bile acid receptor FXR Mol Cell 2003 11 4 1079 1092 10.1016/S1097-2765(03)00104-7 12718892 PMC6179153

[B12] Thomas C Pellicciari R Pruzanski M Auwerx J Schoonjans K Targeting bile-acid signalling for metabolic diseases Nat Rev Drug Discov 2008 7 8 678 693 10.1038/nrd2619 18670431

[B13] Musso G Gambino R Cassader M Emerging molecular targets for the treatment of nonalcoholic fatty liver disease Annu Rev Med 2010 61 375 392 10.1146/annurev.med.60.101107.134820 20059344

[B14] Sinal CJ Targeted disruption of the nuclear receptor FXR/BAR impairs bile acid and lipid homeostasis Cell 2000 102 6 731 744 10.1016/S0092-8674(00)00062-3 11030617

[B15] Kong B Luyendyk JP Tawfik O Guo GL Farnesoid X receptor deficiency induces nonalcoholic steatohepatitis in low-density lipoprotein receptor-knockout mice fed a high-fat diet J Pharmacol Exp Ther 2009 328 1 116 122 10.1124/jpet.108.144600 18948497 PMC2685903

[B16] Zhang Y Castellani LW Sinal CJ Gonzalez FJ Edwards PA Peroxisome proliferator-activated receptor-gamma coactivator 1alpha (PGC-1alpha) regulates triglyceride metabolism by activation of the nuclear receptor FXR Genes Dev 2004 18 2 157 169 10.1101/gad.1138104 14729567 PMC324422

[B17] Claudel T Farnesoid X receptor agonists suppress hepatic apolipoprotein CIII expression Gastroenterology 2003 125 2 544 555 10.1016/S0016-5085(03)00896-5 12891557

[B18] Yamagata K Bile acids regulate gluconeogenic gene expression via small heterodimer partner-mediated repression of hepatocyte nuclear factor 4 and Foxo1 J Biol Chem 2004 279 22 23158 23165 10.1074/jbc.M314322200 15047713

[B19] Ma K Saha PK Chan L Moore DD Farnesoid X receptor is essential for normal glucose homeostasis J Clin Invest 2006 116 4 1102 1109 10.1172/JCI25604 16557297 PMC1409738

[B20] Inagaki T Regulation of antibacterial defense in the small intestine by the nuclear bile acid receptor Proc Natl Acad Sci USA 2006 103 10 3920 3925 10.1073/pnas.0509592103 16473946 PMC1450165

[B21] Chakravarthy MV Identification of a physiologically relevant endogenous ligand for Pplpha in liver Cell 2009 138 3 476 488 10.1016/j.cell.2009.05.036 19646743 PMC2725194

[B22] Okamura M Inagaki T Tanaka T Sakai J Role of histone methylation and demethylation in adipogenesis and obesity Organogenesis 2010 6 1 24 32 10.4161/org.6.1.11121 20592862 PMC2861740

[B23] Nissen SE Wolski K Rosiglitazone revisited: an updated meta-analysis of risk for myocardial infarction and cardiovascular mortality Arch Intern Med 2010 170 14 1191 1201 20656674 10.1001/archinternmed.2010.207

[B24] Dongiovanni P Valenti L Peroxisome proliferator-activated receptor genetic polymorphisms and nonalcoholic Fatty liver disease: any role in disease susceptibility? PPAR Research 2013 2013 452061 23431284 10.1155/2013/452061 PMC3575610

[B25] Sahebkar A Does PPARgamma2 gene Pro12Ala polymorphism affect nonalcoholic fatty liver disease risk? Evidence from a meta-analysis DNA Cell Biol 2013 32 4 188 198 10.1089/dna.2012.1947 23448101

[B26] Wang J Association between the Pro12Ala polymorphism of PPAR-gamma gene and the non-alcoholic fatty liver disease: a meta-analysis Gene 2013 528 2 328 334 10.1016/j.gene.2013.07.014 23891820

[B27] Lazarow PB Fujiki Y Biogenesis of peroxisomes Annu Rev Cell Biol 1985 1 489 530 10.1146/annurev.cb.01.110185.002421 3916321

[B28] Wanders RJ Tager JM Lipid metabolism in peroxisomes in relation to human disease Mol Aspects Med 1998 19 2 69 154 9827430 10.1016/s0098-2997(98)00003-x

[B29] Wanders RJ Waterham HR Biochemistry of mammalian peroxisomes revisited Annu Rev Biochem 2006 75 295 332 10.1146/annurev.biochem.74.082803.133329 16756494

[B30] Abe I Okumoto K Tamura S Fujiki Y Clofibrate-inducible, 28-kDa peroxisomal integral membrane protein is encoded by PEX11 FEBS Lett 1998 431 3 468 472 10.1016/S0014-5793(98)00815-1 9714566

[B31] Delille HK Pex11pbeta-mediated growth and division of mammalian peroxisomes follows a maturation pathway J Cell Sci 2010 123 Pt 16 2750 2762 20647371 10.1242/jcs.062109

[B32] Koch J PEX11 family members are membrane elongation factors that coordinate peroxisome proliferation and maintenance J Cell Sci 2010 123 Pt 19 3389 3400 20826455 10.1242/jcs.064907

[B33] Marshall PA Pmp27 promotes peroxisomal proliferation J Cell Biol 1995 129 2 345 355 10.1083/jcb.129.2.345 7721939 PMC2199913

[B34] Schrader M Expression of PEX11beta mediates peroxisome proliferation in the absence of extracellular stimuli J Biol Chem 1998 273 45 29607 29614 10.1074/jbc.273.45.29607 9792670

[B35] Li X PEX11alpha is required for peroxisome proliferation in response to 4-phenylbutyrate but is dispensable for peroxisome proliferator-activated receptor alpha-mediated peroxisome proliferation Mol Cell Biol 2002 22 23 8226 8240 10.1128/MCB.22.23.8226-8240.2002 12417726 PMC134051

[B36] Weng H Pex11alpha deficiency impairs peroxisome elongation and division and contributes to nonalcoholic fatty liver in mice Am J Physiol Endocrinol Metab 2013 304 2 E187 E196 10.1152/ajpendo.00425.2012 23169785

[B37] Ma W Sung HJ Park JY Matoba S Hwang PM A pivotal role for p53: balancing aerobic respiration and glycolysis J Bioenerg Biomembr 2007 39 3 243 246 10.1007/s10863-007-9083-0 17551815

[B38] Yahagi N p53 involvement in the pathogenesis of fatty liver disease J Biol Chem 2004 279 20 20571 20575 10.1074/jbc.M400884200 14985341

[B39] Farrell GC Apoptosis in experimental NASH is associated with p53 activation and TRAIL receptor expression J Gastroenterol Hepatol 2009 24 3 443 452 10.1111/j.1440-1746.2009.05785.x 19226377

[B40] Derdak Z Inhibition of p53 attenuates steatosis and liver injury in a mouse model of non-alcoholic fatty liver disease J Hepatol 2013 58 4 785 791 10.1016/j.jhep.2012.11.042 23211317 PMC3612370

[B41] Castro RE miR-34a/SIRT1/p53 is suppressed by ursodeoxycholic acid in the rat liver and activated by disease severity in human non-alcoholic fatty liver disease J Hepatol 2013 58 1 119 125 10.1016/j.jhep.2012.08.008 22902550

[B42] Pommier AJ Liver x receptors protect from development of prostatic intra-epithelial neoplasia in mice PLoS Genet 2013 9 5 e1003483 10.1371/journal.pgen.1003483 23675307 PMC3649972

[B43] Zelcer N Hong C Boyadjian R Tontonoz P LXR Regulates Cholesterol Uptake Through Idol-Dependent Ubiquitination of the LDL Receptor Science 2009 325 5936 100 104 10.1126/science.1168974 19520913 PMC2777523

[B44] Suckling K Selective thyromimetics for atherosclerosis and dyslipidaemia: another old target making progress Expert Opin Investig Drugs 2008 17 5 615 618 10.1517/13543784.17.5.615 18447589

[B45] Berkenstam A The thyroid hormone mimetic compound KB2115 lowers plasma LDL cholesterol and stimulates bile acid synthesis without cardiac effects in humans Proc Natl Acad Sci USA 2008 105 2 663 667 10.1073/pnas.0705286104 18160532 PMC2206593

[B46] Cable EE Reduction of hepatic steatosis in rats and mice after treatment with a liver-targeted thyroid hormone receptor agonist Hepatology 2009 49 2 407 417 10.1002/hep.22572 19072834

[B47] Grant N The role of triiodothyronine-induced substrate cycles in the hepatic response to overnutrition: thyroid hormone as an antioxidant Med Hypotheses 2007 68 3 641 649 10.1016/j.mehy.2006.07.045 17023119

[B48] Hardie DG AMP-activated protein kinase: an energy sensor that regulates all aspects of cell function Genes Dev 2011 25 18 1895 1908 10.1101/gad.17420111 21937710 PMC3185962

[B49] Barnes K Activation of GLUT1 by metabolic and osmotic stress: potential involvement of AMP-activated protein kinase (AMPK) J Cell Sci 2002 115 Pt 11 2433 2442 12006627 10.1242/jcs.115.11.2433

[B50] Marsin AS Phosphorylation and activation of heart PFK-2 by AMPK has a role in the stimulation of glycolysis during ischaemia Current Biology : CB 2000 10 20 1247 1255 10.1016/S0960-9822(00)00742-9 11069105

[B51] Mihaylova MM Class IIa histone deacetylases are hormone-activated regulators of FOXO and mammalian glucose homeostasis Cell 2011 145 4 607 621 10.1016/j.cell.2011.03.043 21565617 PMC3117637

[B52] Jorgensen SB The alpha2-5'AMP-activated protein kinase is a site 2 glycogen synthase kinase in skeletal muscle and is responsive to glucose loading Diabetes 2004 53 12 3074 3081 10.2337/diabetes.53.12.3074 15561936

[B53] Koo SH The CREB coactivator TORC2 is a key regulator of fasting glucose metabolism Nature 2005 437 7062 1109 1111 10.1038/nature03967 16148943

[B54] Foretz M Short-term overexpression of a constitutively active form of AMP-activated protein kinase in the liver leads to mild hypoglycemia and fatty liver Diabetes 2005 54 5 1331 1339 10.2337/diabetes.54.5.1331 15855317

[B55] Jager S Handschin C St-Pierre J Spiegelman BM AMP-activated protein kinase (AMPK) action in skeletal muscle via direct phosphorylation of PGC-1alpha Proc Natl Acad Sci USA 2007 104 29 12017 12022 10.1073/pnas.0705070104 17609368 PMC1924552

[B56] Narkar VA AMPK and PPARdelta agonists are exercise mimetics Cell 2008 134 3 405 415 10.1016/j.cell.2008.06.051 18674809 PMC2706130

[B57] Jorgensen SB Effects of alpha-AMPK knockout on exercise-induced gene activation in mouse skeletal muscle FASEB J 2005 19 9 1146 1148 15878932 10.1096/fj.04-3144fje

[B58] Zhang W Thiazolidinediones improve hepatic fibrosis in rats with non-alcoholic steatohepatitis by activating the adenosine monophosphate-activated protein kinase signalling pathway Clin Exp Pharmacol Physiol 2012 39 12 1026 1033 10.1111/1440-1681.12020 23127227

[B59] Zhou G Role of AMP-activated protein kinase in mechanism of metformin action J Clin Invest 2001 108 8 1167 1174 11602624 10.1172/JCI13505 PMC209533

[B60] Zang M AMP-activated protein kinase is required for the lipid-lowering effect of metformin in insulin-resistant human HepG2 cells J Biol Chem 2004 279 46 47898 47905 10.1074/jbc.M408149200 15371448

[B61] Garcia-Ruiz I Solis-Munoz P Fernandez-Moreira D Munoz-Yague T Solis-Herruzo JA Pioglitazone leads to an inactivation and disassembly of complex I of the mitochondrial respiratory chain BMC Biol 2013 11 88 10.1186/1741-7007-11-88 23915000 PMC3751493

[B62] Divakaruni AS Thiazolidinediones are acute, specific inhibitors of the mitochondrial pyruvate carrier Proc Natl Acad Sci USA 2013 110 14 5422 5427 10.1073/pnas.1303360110 23513224 PMC3619368

[B63] Baur JA Resveratrol improves health and survival of mice on a high-calorie diet Nature 2006 444 7117 337 342 10.1038/nature05354 17086191 PMC4990206

[B64] Hwang JT Apoptotic effect of EGCG in HT-29 colon cancer cells via AMPK signal pathway Cancer Lett 2007 247 1 115 121 10.1016/j.canlet.2006.03.030 16797120

[B65] Hou X SIRT1 regulates hepatocyte lipid metabolism through activating AMP-activated protein kinase J Biol Chem 2008 283 29 20015 20026 10.1074/jbc.M802187200 18482975 PMC2459285

[B66] Collins QF Epigallocatechin-3-gallate (EGCG), a green tea polyphenol, suppresses hepatic gluconeogenesis through 5'-AMP-activated protein kinase J Biol Chem 2007 282 41 30143 30149 10.1074/jbc.M702390200 17724029 PMC2408735

[B67] Gledhill JR Montgomery MG Leslie AG Walker JE Mechanism of inhibition of bovine F1-ATPase by resveratrol and related polyphenols Proc Natl Acad Sci USA 2007 104 34 13632 13637 10.1073/pnas.0706290104 17698806 PMC1948022

[B68] Valenti D Negative modulation of mitochondrial oxidative phosphorylation by epigallocatechin-3 gallate leads to growth arrest and apoptosis in human malignant pleural mesothelioma cells Biochimica et Piophysica Acta 2013 1832 12 2085 2096 10.1016/j.bbadis.2013.07.014 23911347

[B69] Bergeron R Effect of 5-aminoimidazole-4-carboxamide-1-beta-D-ribofuranoside infusion on in vivo glucose and lipid metabolism in lean and obese Zucker rats Diabetes 2001 50 5 1076 1082 10.2337/diabetes.50.5.1076 11334411

[B70] Taylor EB Discovery of TBC1D1 as an insulin-, AICAR-, and contraction-stimulated signaling nexus in mouse skeletal muscle J Biol Chem 2008 283 15 9787 9796 10.1074/jbc.M708839200 18276596 PMC2442306

[B71] Mukhtar MH Inhibition of glucokinase translocation by AMP-activated protein kinase is associated with phosphorylation of both GKRP and 6-phosphofructo-2-kinase/fructose-2,6-bisphosphatase Am J Physiol Regul Integr Comp Physiol 2008 294 3 R766 R774 10.1152/ajpregu.00593.2007 18199594

[B72] Guigas B AMP-activated protein kinase-independent inhibition of hepatic mitochondrial oxidative phosphorylation by AICA riboside Biochem J 2007 404 3 499 507 10.1042/BJ20070105 17324122 PMC1896274

[B73] Sanders MJ Grondin PO Hegarty BD Snowden MA Carling D Investigating the mechanism for AMP activation of the AMP-activated protein kinase cascade Biochem J 2007 403 1 139 148 10.1042/BJ20061520 17147517 PMC1828883

[B74] Guigas B Beyond AICA riboside: in search of new specific AMP-activated protein kinase activators IUBMB life 2009 61 1 18 26 10.1002/iub.135 18798311 PMC2845387

[B75] Song Z Involvement of AMP-activated protein kinase in beneficial effects of betaine on high-sucrose diet-induced hepatic steatosis Am J Physiol Gastrointest Liver Physiol 2007 293 4 G894 G902 10.1152/ajpgi.00133.2007 17702954 PMC4215798

[B76] Lee WJ Alpha-lipoic acid increases insulin sensitivity by activating AMPK in skeletal muscle Biochem Biophys Res Commun 2005 332 3 885 891 10.1016/j.bbrc.2005.05.035 15913551

[B77] Yi X Pashaj A Xia M Moreau R Reversal of obesity-induced hypertriglyceridemia by (R)-alpha-lipoic acid in ZDF (fa/fa) rats. Biochemical and biophysical research communications 2013 http://www.ncbi.nlm.nih.gov/pubmed/?term=Reversal+of+obesity-induced+hypertriglyceridemia+by+(R)-alpha-lipoic+acid+in+ZDF+(fa%2Ffa)+rats 10.1016/j.bbrc.2013.08.063 23994635

[B78] Yamauchi T Targeted disruption of AdipoR1 and AdipoR2 causes abrogation of adiponectin binding and metabolic actions Nat Med 2007 13 3 332 339 10.1038/nm1557 17268472

[B79] Andreelli F Liver adenosine monophosphate-activated kinase-alpha2 catalytic subunit is a key target for the control of hepatic glucose production by adiponectin and leptin but not insulin Endocrinology 2006 147 5 2432 2441 10.1210/en.2005-0898 16455782

[B80] Xu A The fat-derived hormone adiponectin alleviates alcoholic and nonalcoholic fatty liver diseases in mice J Clin Invest 2003 112 1 91 100 12840063 10.1172/JCI17797 PMC162288

[B81] McMahan RH Bile acid receptor activation modulates hepatic monocyte activity and improves nonalcoholic fatty liver disease J Biol Chem 2013 288 17 11761 11770 10.1074/jbc.M112.446575 23460643 PMC3636865

[B82] Bataille AM Manautou JE Nrf2: A Potential Target for New Therapeutics in Liver Disease Clin Pharmacol Ther 2012 92 3 340 348 10.1038/clpt.2012.110 22871994 PMC3704160

[B83] Begriche K Massart J Robin MA Bonnet F Fromenty B Mitochondrial adaptations and dysfunctions in nonalcoholic fatty liver disease Hepatology 2013 58 4 1497 1507 10.1002/hep.26226 23299992

[B84] Garcia-Ruiz C Baulies A Mari M Garcia-Roves PM Fernandez-Checa JC Mitochondrial dysfunction in non-alcoholic fatty liver disease and insulin resistance: Cause or consequence? Free Radic Res 2013 47 11 854 868 10.3109/10715762.2013.830717 23915028

[B85] Koliaki C Roden M Hepatic energy metabolism in human diabetes mellitus, obesity and non-alcoholic fatty liver diseasel Cell Endocrinol 2013 379 1–2 35 42 10.1016/j.mce.2013.06.002 23770462

[B86] Fabbrini E Sullivan S Klein S Obesity and nonalcoholic fatty liver disease: biochemical, metabolic, and clinical implications Hepatology 2010 51 2 679 689 10.1002/hep.23280 20041406 PMC3575093

[B87] Tamura S Shimomura I Contribution of adipose tissue and de novo lipogenesis to nonalcoholic fatty liver disease J Clin Invest 2005 115 5 1139 1142 15864343 10.1172/JCI24930 PMC1087181

[B88] Crescenzo R Increased hepatic de novo lipogenesis and mitochondrial efficiency in a model of obesity induced by diets rich in fructose Eur J Nutr 2013 52 2 537 545 10.1007/s00394-012-0356-y 22543624

[B89] Ciapaite J Differential effects of short- and long-term high-fat diet feeding on hepatic fatty acid metabolism in rats Biochim Biophys Acta 2011 1811 7–8 441 451 21621638 10.1016/j.bbalip.2011.05.005

[B90] Dasarathy S Elevated hepatic fatty acid oxidation, high plasma fibroblast growth factor 21, and fasting bile acids in nonalcoholic steatohepatitis Eur J Gastroenterol Hepatol 2011 23 5 382 388 10.1097/MEG.0b013e328345c8c7 21448070 PMC3493151

[B91] Myers MG Jr Leibel RL Seeley RJ Schwartz MW Obesity and leptin resistance: distinguishing cause from effect Trends Endocrinol Metab 2010 21 11 643 651 10.1016/j.tem.2010.08.002 20846876 PMC2967652

[B92] Dushay J Increased fibroblast growth factor 21 in obesity and nonalcoholic fatty liver disease Gastroenterology 2010 139 2 456 463 10.1053/j.gastro.2010.04.054 20451522 PMC4862867

[B93] Li H Fibroblast growth factor 21 levels are increased in nonalcoholic fatty liver disease patients and are correlated with hepatic triglyceride J Hepatol 2010 53 5 934 940 10.1016/j.jhep.2010.05.018 20675007

[B94] Matthews VB Interleukin-6-deficient mice develop hepatic inflammation and systemic insulin resistance Diabetologia 2010 53 11 2431 2441 10.1007/s00125-010-1865-y 20697689

[B95] Takamura T Obesity upregulates genes involved in oxidative phosphorylation in livers of diabetic patients Obesity 2008 16 12 2601 2609 10.1038/oby.2008.419 18846047

[B96] Alves TC Regulation of hepatic fat and glucose oxidation in rats with lipid-induced hepatic insulin resistance Hepatology 2011 53 4 1175 1181 10.1002/hep.24170 21400553 PMC3077048

[B97] Esposito E Probiotics reduce the inflammatory response induced by a high-fat diet in the liver of young rats J Nutr 2009 139 5 905 911 10.3945/jn.108.101808 19321579

[B98] Serviddio G Alterations of hepatic ATP homeostasis and respiratory chain during development of non-alcoholic steatohepatitis in a rodent model Eur J Clin Invest 2008 38 4 245 252 10.1111/j.1365-2362.2008.01936.x 18339004

[B99] Lazarin Mde O Liver mitochondrial function and redox status in an experimental model of non-alcoholic fatty liver disease induced by monosodium L-glutamate in rats Exp Mol Pathol 2011 91 3 687 694 10.1016/j.yexmp.2011.07.003 21821020

[B100] Flamment M Regulation of hepatic mitochondrial metabolism in response to a high fat diet: a longitudinal study in rats J Physiol Biochem 2012 68 3 335 344 10.1007/s13105-012-0145-3 22278845

[B101] Jiang Y Zhang H Dong LY Wang D An W Increased hepatic UCP2 expression in rats with nonalcoholic steatohepatitis is associated with upregulation of Sp1 binding to its motif within the proximal promoter region J Cell Biochem 2008 105 1 277 289 10.1002/jcb.21827 18543254

[B102] Begriche K Massart J Robin MA Borgne-Sanchez A Fromenty B Drug-induced toxicity on mitochondria and lipid metabolism: mechanistic diversity and deleterious consequences for the liver J Hepatol 2011 54 4 773 794 10.1016/j.jhep.2010.11.006 21145849

[B103] Mingorance C Propionyl-L-carnitine corrects metabolic and cardiovascular alterations in diet-induced obese mice and improves liver respiratory chain activity PloS one 2012 7 3 e34268 10.1371/journal.pone.0034268 22457831 PMC3311627

[B104] Finocchietto PV Defective leptin-AMP-dependent kinase pathway induces nitric oxide release and contributes to mitochondrial dysfunction and obesity in ob/ob mice Antioxid Redox Signal 2011 15 9 2395 2406 10.1089/ars.2010.3857 21529143

[B105] Pessayre D Role of mitochondria in non-alcoholic fatty liver disease J Gastroenterol Hepatol 2007 22 Suppl 1 S20 S27 17567459 10.1111/j.1440-1746.2006.04640.x

[B106] Mollica MP 3,5-diiodo-l-thyronine, by modulating mitochondrial functions, reverses hepatic fat accumulation in rats fed a high-fat diet J Hepatol 2009 51 2 363 370 10.1016/j.jhep.2009.03.023 19464748

[B107] Hancock CR High-fat diets cause insulin resistance despite an increase in muscle mitochondria Proc Natl Acad Sci USA 2008 105 22 7815 7820 10.1073/pnas.0802057105 18509063 PMC2409421

[B108] Ramirez-Tortosa MC Curcumin ameliorates rabbits's steatohepatitis via respiratory chain, oxidative stress, and TNF-alpha Free Radic Biol Med 2009 47 7 924 931 10.1016/j.freeradbiomed.2009.06.015 19539747

[B109] Serviddio G A silybin-phospholipid complex prevents mitochondrial dysfunction in a rodent model of nonalcoholic steatohepatitis J Pharmacol Exp Ther 2010 332 3 922 932 10.1124/jpet.109.161612 20008062

[B110] Samuhasaneeto S Thong-Ngam D Kulaputana O Patumraj S Klaikeaw N Effects of N-acetylcysteine on oxidative stress in rats with non-alcoholic steatohepatitis J Med Assoc Thai 2007 90 4 788 797 17487136

[B111] Hezode C Daily cannabis smoking as a risk factor for progression of fibrosis in chronic hepatitis C Hepatology 2005 42 1 63 71 10.1002/hep.20733 15892090

[B112] Munoz-Luque J Regression of fibrosis after chronic stimulation of cannabinoid CB2 receptor in cirrhotic rats J Pharmacol Exp Ther 2008 324 2 475 483 18029545 10.1124/jpet.107.131896 PMC2887659

[B113] Teixeira-Clerc F CB1 cannabinoid receptor antagonism: a new strategy for the treatment of liver fibrosis Nat Med 2006 12 6 671 676 10.1038/nm1421 16715087

[B114] Van Gaal L Pi-Sunyer X Despres JP McCarthy C Scheen A Efficacy and safety of rimonabant for improvement of multiple cardiometabolic risk factors in overweight/obese patients: pooled 1-year data from the Rimonabant in Obesity (RIO) program Diabetes Care 2008 31 Suppl 2 S229 S240 18227491 10.2337/dc08-s258

[B115] Janero DR Lindsley L Vemuri VK Makriyannis A Cannabinoid 1 G protein-coupled receptor (periphero-)neutral antagonists: emerging therapeutics for treating obesity-driven metabolic disease and reducing cardiovascular risk Expert Opinion on Drug Discovery 2011 6 10 995 1025 10.1517/17460441.2011.608063 22646861

